# Bacteria

**Published:** 1881-10

**Authors:** S. O. Gleason


### BACTERIA.

---

S. O. GLEASON, M. D.

---

When a fluid in which there are organic substances, remains in warm air for some time, changes take place which are called putrefactive or fermentative. There is usually also a slight liberation of gas from the infusion, then the fluid becomes more turbid and a partially transparent scum or pellicle forms on the top of the same; this is called by Burdach—the "primordial mucous layer" or by Pouchet the "proligerous pellicle."

When this is examined under the highest microscopic power, multitudes of active specks will be seen in rapid motion. Some will appear spherical and others staff-like, known and recognized as Bacteria.

The small minute specks from the $\frac{1}{20000}$ to the $\frac{1}{100000}$ of an inch in diameter, are now called *plastide-particles.* They are recognized by Bastian and many other observers as the primordial particles of *living matter.* I have seen the above mentioned plastide-particles and Bacteria, and also other minute organisms in various infusions.

Various opinions are held in regard to the origin and nature of Bacteria. By some they are supposed to be the result of the aggregation of homogenous plastide-particles.

The longer and more developed bodies called *bibriones* have been thought to be, by some, the result of the coalescence of Bacteria. Bastian thinks it more probable that both Bacteria and bibriones are only the result of the more perfect growth and development of the simple, minute plastide-particles of living matter.

Dr. Bennett thinks he has seen the actual coalescence of such particles. I do not know of any other observer who makes any claim to have witnessed this phenomenon. So, we still have discordant opinions as to the real nature of Bacteria.

Naturalists are still in doubt whether they are "independent living things" of a low order, or whether they have a distinct individuality of themselves, or whether they are but the simple forms of life, capable of being developed into higher living things—either animal or vegetable. There are four distinct opinions in regard to them. I quote from Bastian the following : "First, they are animal organisms of the lowest grade having a special individuality as conjectured by Erhenberg. Second, they are thought by Hallier to be of the nature of spores, produced from, and destined again to develop into, some of the simplest microscopic fungi. Third, that they represent, as Cohn thinks, the later free-swimming stage in the existence of certain Algæ, intermediate between Palmella and Oscillatoria. Fourth, that they are the first and most common developmental phase of newly evolved specks of living matter, which are capable, either singly or in combination, of developing into many kinds of living things.

Bacteria are not all of the same size or shape. They vary in the kinds of movements they make. They seem to vary according to the putrescent quality of the infusion, and the degree of heat to which it has been exposed, also other modifying conditions come into play. They are, for the most part, straight rod-like bodies. They vary in length from the $\frac{1}{20000}$ to the $\frac{1}{150000}$ of an inch. A joint or line in the center divides them into equal parts. Their movements are more or less rapid, they oscillate and irregularly rotate —while at times they dart from place to place, either direct, or in variously curved lines.

It should be remembered that such forms of life as the above described, only constitute one variety of the very many lower forms of living things found in organic solutions. The one form described is perhaps the most usual one met with in infusions.

We find other bi-segmented bodies less cylindrical—which are not rigid, but have a flexible joint so that the two parts move freely.

There are other forms that are bicellular in appearance. One other form resembles the figure eight—seeming to be made of partial cells. Others are neck-lace like and composed of from two to fifteen bodies, about the size of plastide-particles, but they are more hollow in appearance. Organisms are also seen which present the appearance of the small vegetable cells of the great fungus. All who work much with the microscope will recognize these and many other minute and interesting forms of life. The microscope takes us to the dim shadowy boundaries of life, inspiring us with fresh zeal and to new endeavors to come nearer the grand, sublime and awful mysteries of life.

Have you paid your subscription to the BISTOURY? If not, remit before you forget it.

### DIPHTHERIA AND SCARLET FEVER.

The following extract is from a circular issued by Dr. James Crane of the Brooklyn Health Department :

Diphtheria and scarlet fever are highly contagious diseases, attacking persons of all ages. They may be contracted from persons that are already affected, from the clothes they have worn and from everything which has been in the room with them. Even the walls of the room may infect persons coming into it after the patient has recovered, unless the poison is destroyed. In order to prevent their spread in the family or house where they exist, and to promote the recovery of the persons attacked the following simple measures should be conscientiously and rigidly carried out :—An upper, sunny room, provided if possible sible with an open fireplace, and with no other children on the same floor, should be arranged for the patient by removing everything from it which can possibly be spared, such as books, clothing and window curtains, remembering that when once the patient has entered the room nothing can with safety be removed until disinfected or fumigated. One or two adults should have entire charge of the patient, under no circumstances coming in contact with other persons, more especially children. Open windows and open fireplaces, with fire in them day and night, avoiding draughts and chilly air, protect the sick and those who nurse them.

Put into an ordinary water pail eight tablespoonsfuls of sulphate of zinc and four of common salt, and to this add one gallon of boiling water. This disinfecting solution is to be kept in the room, and into it should be placed and kept for one hour every article of soiled clothing, bedding, handkerchiefs, &c. When they are removed from this they should be put into boiling water before being washed. The dishes and spoons used by the patient should be put into boiling water before they are permitted to leave the room. Remember that every article which is in the room can convey the disease and that nothing should go from it until the poison which it might carry is destroyed.

Keep the cellar dry, well ventilated and well whitewashed, never allow even for a day, garbage or other filth to be kept in it. Open the window of the sleeping rooms every day for as long a time as possible, fresh air being an excellent disinfectant.

